# Erythrodermic Psoriasis Exacerbated by Bupropion

**DOI:** 10.7759/cureus.18460

**Published:** 2021-10-03

**Authors:** Michael G Foss, Timothy Nyckowski, William Steffes

**Affiliations:** 1 Dermatology, Kansas City University-Graduate Medical Education Consortium/Advanced Dermatology and Cosmetic Surgery Orlando Dermatology Residency Program, Orlando, USA

**Keywords:** erythrodermic psoriasis, drug rash, drug reaction, bupropion, erythroderma, psoriasis

## Abstract

Erythroderma is a rare, potentially life-threatening presentation of psoriasis that can be triggered by medication reactions. Bupropion is indicated for major depressive disorder (Wellbutrin®, GlaxoSmithKline, Research Triangle Park, NC), smoking cessation (Zyban®, GlaxoSmithKline, Research Triangle Park, NC), and weight loss (when in formulation with naltrexone ER; Contrave®, Orixegen Therapeutics, La Jolla, CA). Bupropion can exacerbate psoriasis, however, this is an under-recognized side effect of the medication, particularly in the United States. We report a case of bupropion-induced erythrodermic psoriasis in a 62-year-old female who was prescribed the medication for depression. Due to the common comorbidities of depression, obesity, and tobacco abuse in psoriatic patients, all for which treatment with bupropion is indicated, it is important for physicians to be aware of the potential for a life-threatening medication reaction in this patient population.

## Introduction

Psoriasis affects up to 4.6% of the United States (US) population, and up to 2.25% of psoriatic patients may develop the erythrodermic variant, involving >80% of the body surface area (BSA) [1-4]. We report a case of a 62-year-old female with an acute eruption of erythrodermic psoriasis, four days after initiation of bupropion, a popularly prescribed antidepressant (Wellbutrin®, GlaxoSmithKline, Research Triangle Park, NC), smoking cessation aid (Zyban®, GlaxoSmithKline, Research Triangle Park, NC), and weight loss adjunct (when in combination with naltrexone ER; Contrave®, Orixegen Therapeutics, La Jolla, CA). Though rare, there have been increasing reports of bupropion inducing erythrodermic psoriasis, and the clinician should be aware of this association and elicit a history of psoriasis when prescribing this medication.

## Case presentation

A 62-year-old female was brought to the hospital emergency department by ambulance for a diffuse skin eruption. Four days prior to presentation, she was initiated on bupropion for depression, which was previously treated with mirtazapine. Her medication list also included clobetasol ointment, calcipotriene ointment, levothyroxine, metformin, and mirabegron. She had a long-standing history of psoriasis affecting her scalp, extensor extremities, lower back, and chest. Additional medical history included hypothyroidism, diabetes, and urinary incontinence. 

**Figure 1:**
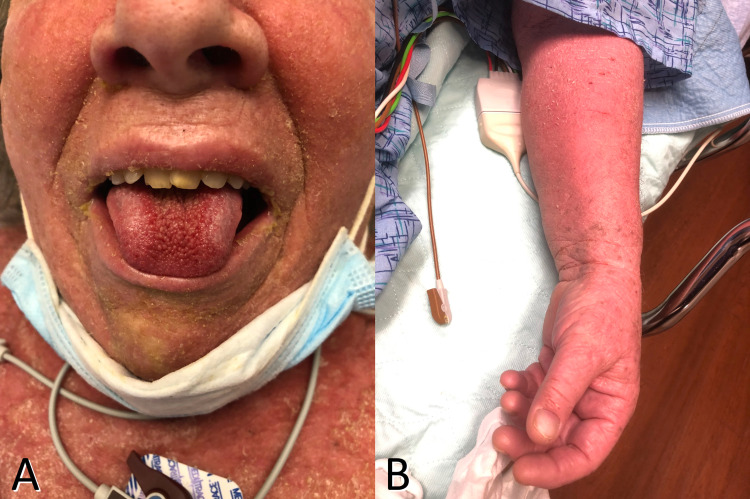
Erythrodermic psoriasis Diffuse erythema and scaling involving >80% of the patient’s body surface area (A and B), with concomitant mucosal changes consistent with geographic tongue (A).

Two days after beginning bupropion, her psoriasis spread to involve flexural skin areas, with a further spread in the following two days. On day four, she presented to the hospital because her psoriasis covered the majority of her body surface, accompanied by mucosal changes, subjective fever, and fatigue (Figure 1).

**Figure 2:**
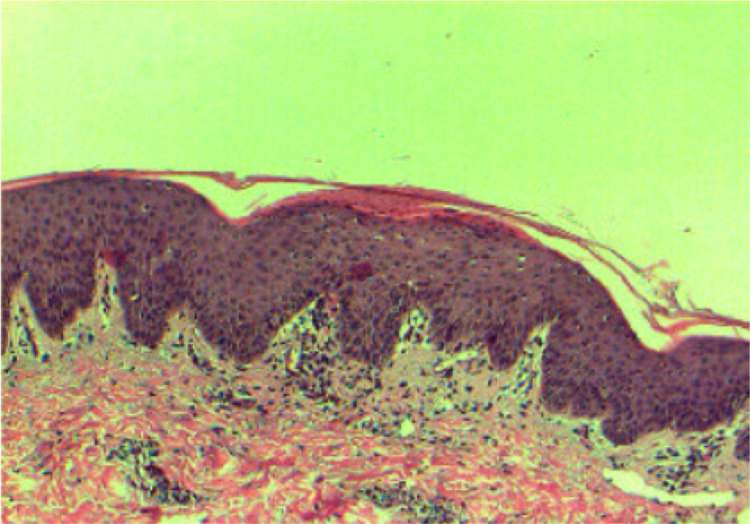
Erythrodermic psoriasis (H&E original magnification x 100) Hyperkeratosis, parakeratosis, and acanthosis with elongation of rete ridges. Neutrophils are present in the stratum corneum, forming Munro microabscesses. The papillary dermis is edematous with dilated capillaries.

Upon arrival to the emergency room, she was found to be tachycardic, tachypneic, hypotensive, and febrile. Laboratory evaluation revealed a leukocytosis of 22 x10^9^/L (reference range: 4.5-11.0 × 10^9^/L) and an elevated creatinine of 1.74 mg/dL (reference range: 0.60-1.10 mg/dL), consistent with acute kidney injury, prompting an initial concern for sepsis along with erythroderma. Dermatology was consulted for further assessment. After obtaining relevant history, performing physical examination, and eliminating the possibility of other potential causes of erythroderma, a provisional diagnosis of bupropion-induced erythrodermic psoriasis was made. Biopsy confirmed the initial clinical diagnosis, showing hyperkeratosis, parakeratosis, and neutrophils in the stratum corneum forming Munro microabscesses (Figure 2).

Bupropion was promptly discontinued. Due to improvement in renal function following fluid resuscitation, the patient was begun on cyclosporine 2.5 mg/kg/day. Her erythroderma cleared within five days, and she followed up in our outpatient clinic for further long-term management including transitioning from cyclosporine to secukinumab, with near resolution of her psoriasis at five-week follow-up.

## Discussion

Erythroderma is generalized erythema and scale involving >80% of the body surface area that can be life-threatening due to systemic manifestations of impaired skin barrier [3,5]. Psoriasis is the most commonly identified trigger of erythroderma, causing up to 25% of cases [3]. It is important to recognize and address the causes of erythrodermic psoriasis, including rapid withdrawal of systemic immunosuppressive therapy, underlying systemic infections, or drug reactions [3,6]. This is the fourth report and sixth case of bupropion-associated erythrodermic psoriasis [1,7,8]. In addition to erythroderma, Stevens-Johnson syndrome and morbilliform drug eruption have also occurred following bupropion initiation, both with concomitant psoriasis exacerbation secondary to koebnerization [9,10].

Bupropion causes reuptake inhibition of both norepinephrine and dopamine, without affecting serotonin [11]. Initially approved in 1985 for major depressive disorder, it has since received approval for seasonal affective disorder and smoking cessation [11-13]. Psychiatrists prescribe bupropion for depression with symptoms of lethargy, sexual dysfunction, and/or weight gain due to its unique side effect profile [11]. Bupropion may even lead to weight loss, an indication for which it was re-branded in combination with naltrexone ER in 2014 [11,14]. 

Minor dermatologic side effects including urticarial and morbilliform rashes are not uncommon with bupropion use, occurring in up to 10% of users [12,13]. Commonly reported serious cutaneous adverse reactions associated with bupropion, although relatively rare, include angioedema and serum-sickness-like reactions [15]. Flaring of psoriasis is an under-recognized side effect of bupropion, particularly in the United States. The manufacturer for bupropion reports worsening of psoriasis among 1 in 1000 people in the United Kingdom (UK) patient leaflet, but this information is conspicuously absent in the US prescribing information [12,13,16].

Psoriasis is a multisystem inflammatory disorder with a number of related comorbidities relevant to populations treated with bupropion, including obesity, cigarette smoking, and depression [17,18]. Psoriasis, obesity, and mood changes are comorbidities unified by elevated tumor necrosis factor (TNF)-alpha levels that may result in a self-amplifying feedback loop [19,20]. This cascade poses an attractive target for treatment with bupropion given its comparative weight-loss properties versus other anti-depressants that can lead to weight gain [11]. Although it may seem logical to prescribe bupropion in the depressed, obese, psoriatic smoker, we suggest doing so with caution. In patients who are candidates for bupropion treatment, we recommend eliciting a history of psoriasis, accompanied by physical examination with attention to cutaneous findings. If bupropion is ultimately prescribed, psoriatic patients should be counseled on the potential for erythroderma and immediate discontinuation if these symptoms occur.

## Conclusions

We report the sixth case of erythrodermic psoriasis related to bupropion initiation. With obesity and depressive disorders in the United States steadily rising, and the recent FDA indication for weight loss, bupropion prescriptions will likely parallel this increase. Prescribers should be aware of the relationship between bupropion and psoriasis, including the dangerous erythrodermic variant. Ultimately, prescribers should take a thorough history of psoriasis and if they confirm, then counsel the patient to immediately stop therapy if they notice significant worsening or spreading of the disease.
